# Tractile Method in Strangulated Hernia

**Published:** 1874-08

**Authors:** Daniel Leasure


*AMERICAN JOURNAL OF THE MEDICAL SCIENCES—April,* 1874.

TRACTILE METHOD OF REDUCING STRANGULATED HERNIA.—*By Daniel Leasure, M.D.*—In the summer of 1843 I was associated with the late Dr. Oliver Cunningham, of Beaver, in the treatment of a case of strangulated femoral hernia, the result of which has since influenced my treatment of that lesion. The patient was a woman, forty years of age, the mother of several children, of robust health till the occurrence of the hernia. In lifting a tub of wet clothes she felt the protrusion take place, which was followed immediately by pain, nausea and vomiting. A messenger was dispatched for Dr. Cunningham, and I being in the neighborhood, was also called in. The pain was almost constant, and the matter vomited stercoraceous.

After persistent and futile efforts at reduction, under the use of morphia and tobacco (there being no anæsthetics in those days), Dr. Cunningham went home, a distance of eight miles, for instruments with which to operate, leaving me in charge of the case, with instructions to keep the patient well under the influence of morphia, and to apply hot fomentations to the hernial tumor. Meantime night came on, and the patient's sufferings became, if that were possible, more intense, until at last she prayed for ease or death. Made desperate by her cries, and hardly knowing what more to try, I stated to her husband that, if he would suspend her by her limbs, with her head downwards, I would make another attempt to push back the protruded bowel. Turning his back to the bed, he "hunkered down," and placing one of the patient's legs over each of his shoulders, as far as the hams, he seized an ankle in each hand, and drew her heels against his breast; then, raising himself to the erect position, the patient hung down along his back, until only her head and shoulders rested on the bed. The patient's abdomen was pendulous, with the abdominal walls overhanging the brim of the pelvis in the erect position, but on being reversed, the belly fell towards the chest. Placing my right hand over the tumor, which was as large as a goose's egg and very tense, and the fingers and thumb of the left hand over its neck; while pausing to think what next to do, the tumor suddenly slipped a little, and in another instant, with the characteristic gurgle, shot away from my touch into the abdominal cavity. The patient was laid on her bed, and in two minutes was soundly sleeping.

